# Extrapericardial tamponade following blunt chest trauma treated with subxiphoid uniportal video-assisted thoracoscopic surgery: a case report

**DOI:** 10.1186/s13019-026-03871-x

**Published:** 2026-02-06

**Authors:** Nahyeon Lee, Seok Hwa Youn, Younghwan Kim, Mina Kim, Jinho Jheong, Gaesung Ha, Youngwoong Kim

**Affiliations:** 1 Division of Thoracic and Cardiovascular Surgery, Department of Trauma Surgery, Seoul Regional Trauma Center, National Medical Center, Seoul, Republic of Korea; 2 Division of Surgery, Department of Trauma Surgery, Seoul Regional Trauma Center, National Medical Center, Seoul, Republic of Korea

**Keywords:** Cardiac tamponade, Thoracic surgery, Video-Assisted, Wounds, Nonpenetrating

## Abstract

**Background:**

Anterior mediastinal hematomas following blunt chest trauma are usually not life threatening. However, in rare cases, they can cause extrapericardial tamponade, requiring urgent surgical intervention. Despite its increasing adoption in elective thoracic procedures, the use of subxiphoid uniportal video-assisted thoracoscopic surgery (SUVATS) in trauma remains scarcely reported. This case highlights a rare instance of extrapericardial tamponade caused by an anterior mediastinal hematoma, successfully managed using SUVATS in a trauma setting.

**Case presentation:**

We report the case of a 65-year-old man who had a 4-meter fall and sustained a retrosternal hematoma and sternal fracture, as confirmed on chest computed tomography without evidence of intracardiac or major vascular injuries. Following initial hemodynamic stability, evolving hemodynamic compromise led to transthoracic echocardiography, which demonstrated right ventricular compression consistent with extrapericardial tamponade, necessitating surgical evacuation. SUVATS was performed for hematoma evacuation and hemostasis. During subsequent sternal fixation via a separate limited midline incision, thoracoscopic visualization enabled assessment of the posterior sternal surface. The patient was extubated on the day of surgery and was discharged without complications.

**Conclusions:**

This case demonstrates the extended utility of SUVATS beyond hematoma evacuation, including its adjunctive role in sternal fixation. This minimally invasive approach provided excellent mediastinal exposure, reduced postoperative pain, and minimized respiratory complications. SUVATS may be a feasible option in selected trauma cases, including those with tamponade physiology. In trauma patients without major vascular injury, it may offer a less invasive yet effective alternative to median sternotomy, potentially improving early recovery and outcomes.

## Background

Blunt chest trauma can lead to anterior mediastinal hematoma due to injuries such as rib/sternum fractures or injuries to the internal mammary artery, intercostal arteries, or mediastinal vessels [1, 2]. Although not all anterior mediastinal hematomas are life threatening, they may occasionally compress cardiac chambers, leading to hemodynamic compromise and necessitating urgent surgical intervention [1–3]. While video-assisted thoracoscopic surgery (VATS) is widely accepted in thoracic procedures, the application of subxiphoid uniportal video-assisted thoracoscopic surgery (SUVATS) in the setting of trauma is rarely reported, particularly for managing anterior mediastinal hematomas with concurrent sternal fractures. This case report aims to illustrate the potential role of SUVATS, not only for hematoma evacuation but also as an adjunct in facilitating sternal stabilization, demonstrating its broader utility in selected trauma scenarios.

## Case presentation

**Fig. 1:**
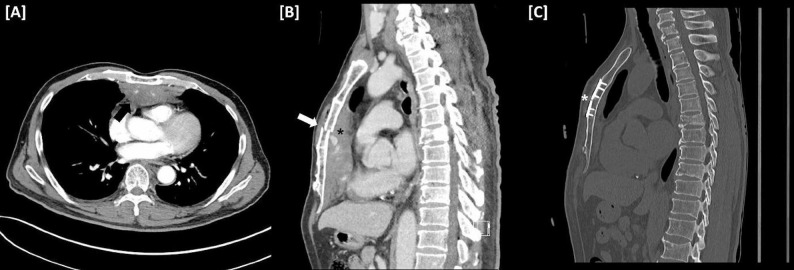
Chest CT scans. [**A**] Preoperative axial images showing an anterior mediastinal hematoma (black arrow). [**B**] Sagittal reconstruction demonstrating a displaced sternum (white arrow) with retrosternal hemorrhage (black asterisk). [**C**] Postoperative follow-up CT showing proper sternal alignment (white asterisk) and resolution of the hematoma

A 65-year-old man with a history of hypertension sustained a 4-meter fall. On admission, his blood pressure was 138/83 mmHg, heart rate 104 beats per minute (BPM), respiratory rate 26 breaths per minute, and oxygen saturation 99%. Chest computed tomography (CT) revealed a sternal fracture and a 10.3 × 3.0 × 8.0 cm retrosternal hematoma (Fig. 1A), without cardiac or major vascular injury. Thirty minutes later, his condition deteriorated, with a blood pressure of 80/50 mmHg and a heart rate of 120 BPM, suggesting cardiac tamponade. Transthoracic echocardiography (TTE) revealed right ventricular compression by an anterior mediastinal hematoma without intracardiac injury.

**Fig. 2:**
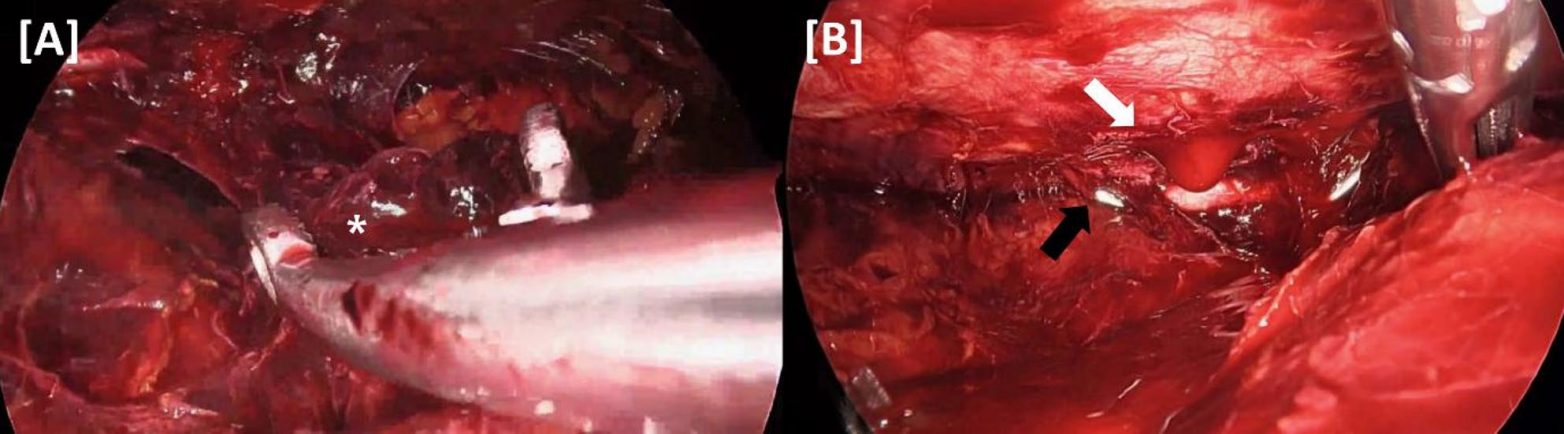
Intraoperative views during SUVATS. [**A**] Hematoma (white asterisk) is evacuated via the subxiphoid approach. [**B**] Active bleeding from the sternal periosteum (white arrow) is identified and controlled. Sternal reduction is performed using bone-reducing forceps (black arrow)

Given the fractured sternum and thymic vessel as potential bleeding sources, surgical intervention was planned to evacuate the hematoma and stabilize the sternum fracture (Fig. 1B). A 2-cm subxiphoid incision was made, and a port was placed. After evacuation of the hematoma, active bleeding from the periosteum surrounding the fractured sternum and multifocal sites within the thymic tissue was controlled using electrocautery (Fig. 2A). No additional vessel injuries were observed. A separate 4-cm midline incision was performed for sternal fixation. Thoracoscopic assistance facilitated visualization of the posterior aspect of the fracture, ensuring accurate reduction and fixation (Fig. 2B). The operative time was 127 min, and the estimated blood loss was 100 mL. Continuous arterial-line monitoring and end-tidal carbon dioxide were used throughout the procedure. Postoperative analgesia consisted of scheduled NSAIDs and acetaminophen.

The patient was transferred to a general ward on postoperative day (POD) 1. A CT scan on POD 6 showed proper sternal alignment without residual hematoma (Fig. 1C). Follow-up on POD 30 showed well-healed wounds, no chest discomfort, and full respiratory recovery without complications.

### Discussion and conclusions

Extrapericardial tamponade resulting from anterior mediastinal hematomas is exceedingly uncommon, and in the absence of cardiac or major vessel injuries, such hematomas only infrequently compress the cardiac chambers following blunt chest trauma [3], making timely diagnosis challenging. CT is valuable for identifying vascular injuries, and when tamponade is suspected, TTE assesses physiological impact and excludes intracardiac injuries [3]. Surgical evacuation is necessary if tamponade is confirmed [3].

The selection of surgical approach must consider both the operative modality (open versus minimally invasive) and the anatomical route (subxiphoid versus transthoracic). Although median sternotomy remains the standard approach for anterior mediastinal hematomas with hemodynamic compromise, this patient met several conditions favoring a minimally invasive strategy. Hemodynamic deterioration occurred gradually rather than precipitously, and bedside TTE demonstrated preserved cardiac function without major vascular injury, indicating that immediate open conversion was not mandatory [2]. In addition, the hematoma was predominantly retrosternal, where the subxiphoid route provides direct, magnified visualization of potential bleeding sources. An emergency sternotomy setup was fully prepared at the bedside, allowing immediate conversion without patient repositioning if uncontrollable hemorrhage occurred. The concurrent transverse sternal fracture also informed the choice of approach. Performing a sternotomy through an already displaced transverse fracture risks irregular bone approximation, instability, and nonunion [4]. Thoracoscopic visualization of the posterior sternal cortex facilitated precise reduction and fixation, which would have been more difficult after dividing the sternum. Furthermore, the subxiphoid approach avoids intercostal incisions, reduces postoperative respiratory impairment, and allows sternal stabilization in the supine position [2, 5].

Despite these advantages, SUVATS has inherent limitations. The visual field is narrower than that of sternotomy, and control of diffuse retrosternal bleeding may be more challenging, especially in polytrauma patients with multiple potential bleeding sites. The subxiphoid route also does not permit full thoracic exploration, creating a risk of missed associated injuries. SUVATS requires substantial thoracoscopic expertise and should not be attempted in profoundly unstable patients or when major vascular injury is suspected. In particular, it should be reserved for surgeons with extensive experience in minimally invasive and subxiphoid approaches, typically acquired in the elective thoracic surgery setting. For these reasons, careful patient selection and readiness for immediate conversion are essential.

This case suggests that SUVATS may be considered in highly selected scenarios: hemodynamically responsive patients without major vascular injury, isolated anterior mediastinal hematoma, and settings where trauma-trained thoracoscopic surgeons and immediate open conversion capability are available. This report does not aim to establish superiority of SUVATS but to illustrate its feasibility and specific advantages in a highly selected trauma setting. Although no comparative studies exist in trauma populations, minimally invasive mediastinal approaches have been associated with reduced postoperative pain, earlier mobilization, and fewer respiratory complications compared with sternotomy in selected thoracic conditions.

In conclusion, anterior mediastinal hematomas, although rare, can cause extrapericardial tamponade, which requires prompt diagnosis and intervention. This case demonstrates that SUVATS can be a viable minimally invasive alternative to sternotomy in appropriately chosen patients, particularly when mediastinal access and concurrent sternal stabilization are required. With appropriate patient selection and surgical expertise, SUVATS may offer improved visualization, reduced pain, and fewer respiratory complications compared to open surgery.

## Data Availability

No datasets were generated or analysed during the current study.

## Abbreviations

VATS: Video-assisted thoracic surgery
SUVATS: Subxiphoid uniportal video-assisted thoracoscopic surgery
BPM: Beats per minute
CT: Computed tomography
TTE: Transthoracic echocardiography
POD: Postoperative day
